# Effects of Feeding of Two Potentially Probiotic Preparations from Lactic Acid Bacteria on the Performance and Faecal Microflora of Broiler Chickens

**DOI:** 10.1100/2012/562635

**Published:** 2012-05-15

**Authors:** Paula Fajardo, Lorenzo Pastrana, Jesús Méndez, Isabel Rodríguez, Clara Fuciños, Nelson P. Guerra

**Affiliations:** ^1^Departamento de Química Analítica y Alimentaria, Facultade de Ciencias de Ourense, Universidade de Vigo, As Lagoas s/n, 32004 Ourense, Spain; ^2^Cooperativas Orensanas Sociedad Cooperativa Ltda (COREN), Polígono San Ciprián de Viñas, 32901 Ourense, Spain

## Abstract

The aim of this study was to evaluate the potential of two probiotic preparations, containing live lactic acid bacteria (*Lactococcus lactis* CECT 539 and *Lactobacillus casei* CECT 4043) and their products of fermentation (organic acids and bacteriocins), as a replacement for antibiotics in stimulating health and growth of broiler chickens. The effects of the supplementation of both preparations (with proven probiotic effect in weaned piglets) and an antibiotic (avilamycin) on body weight gain (BWG), feed intake (FI), feed consumption efficiency (FCE), relative intestinal weight, and intestinal microbiota counts were studied in 1-day posthatch chickens. The experiments were conducted with medium-growth Sasso X44 chickens housed in cages and with nutritional stressed Ross 308 broiler distributed in pens. Consumption of the different diets did not affect significantly the final coliform counts in Sasso X44 chickens. However, counts of lactic acid bacteria and mesophilic microorganisms were higher in the animals receiving the two probiotic preparations (*P* < 0.05). In the second experiment, although no differences in BWG were observed between treatments, Ross 308 broilers receiving the probiotic *Lactobacillus* preparation exhibited the lowest FCE values and were considered the most efficient at converting feed into live weight.

## 1. Introduction

Antibiotics have been extensively used in animal feed to improve production in poultry and piglet industries [1]. However, the use of these substances as growth promoters can lead to the development of antibiotic resistances. Such resistances can occur not only in pathogenic bacteria [2, 3], which can be transferred from poultry products to human population [4], but also in commensal bacteria [5], constituting a reservoir of resistance genes for pathogenic bacteria [6]. In recent years, the interest in finding alternatives to the use of antibiotics in animal feed has been increased due to the ban of subtherapeutic antibiotic usage in Europe. The research is mostly focused on incorporating into animal feeds, substances derived from plants, animals, bacteria and fungi, as well as organic acid, essential oils, and bacteriocins, that could interfere in colonisation of pathogens [7–9].

Due to their potential to reduce enteric disease in poultry, probiotics are considered to be a good alternative to the use of antibiotics [10]. The production of antimicrobial compounds (mainly organic acids and bacteriocins) by many lactic acid bacteria (LAB) into the intestine has provided these organisms with a competitive advantage over other microorganisms to be used as probiotics [11, 12]. Moreover, the presence of some *Lactobacillus *in the chicken gastrointestinal tract (GIT) has been described to be of great importance for regulating the composition of the intestinal microflora, developing immunity of the intestine, and promoting the health of chickens [13].

The administration of highly concentrated bacterial cultures, containing both the live cells and their products of fermentation, was an effective way to promote body weight gain (BWG) and improve feed conversion efficiency (FCE) in chickens [6, 14–16]. In fact, probiotics are used nowadays by compound feed industry to improve the poultry production [17, 18].

In a previous work [1], two potentially probiotic preparations, containing the live cells of *Lactococcus lactis *CECT 539 or *Lactobacillus casei *CECT 4043, as well as their fermentation products, were evaluated as probiotic additives to replace antibiotics in weaning pig diets. The administration of these potentially probiotic preparations improved BWG and feed intake (FI). In the same study, Guerra et al. [1] observed that the two above-mentioned LAB fulfil many of the probiotic criteria [19], because they are (i) nonpathogenic, (ii) able to survive during processing and storage, (iii) resistant to bile and acid environment, and (iv) producer of inhibitory compounds (organic acids and antibacterial activity).

Since probiotic effects are strain dependent [1] and may also depend on the host and their immunologic state [20], the observed probiotic effects of the *L. lactis *CECT 539 and *Lact. casei *CECT 4043 preparations in piglets might not be observed in other host entities. Therefore, in an attempt for testing the latter hypothesis, the *L. lactis *CECT 539 and *Lact. casei *CECT 4043 preparations (containing both the live cells and their products of fermentation) were evaluated as a replacement for antibiotics in stimulating health and growth of broilers.

## 2. Materials and Methods

### 2.1. Bacterial Strains

*Lactobacillus casei *subsp. *casei *CECT 4043 (a high lactic acid-producing strain) and *Lactococcus lactis *subsp. *lactis *CECT 539 (a nisin-producing strain) were obtained from the Spanish Type Culture Collection (CECT). Stock cultures of both LAB strains were maintained at −40°C in nutrient broth supplemented with 15% glycerol. Working cultures, maintained at 4°C on de Man, Rogosa, and Sharpe (MRS, Cultimed) agar, were prepared monthly from frozen stock cultures.

### 2.2. Production of the Potentially Probiotic Preparations of the Two LAB

The potentially probiotic preparations of the strains CECT 4043 and CECT 539 were obtained by using a fed-batch fermentation technique based on successive realkalizations of the culture media, which were prepared with cheese whey from a local dairy plant [21]. The fermentation medium was a diluted whey (DW: concentrated whey (CW) mixed with wash water), which contained (in g/L): total sugars, 20.54; total nitrogen, 0.45; total phosphorus, 0.25, and soluble proteins, 2.04 [1]. The substrates used as feeding media were a concentrated lactose solution (400 g/L) and CW medium. The latter substrate contained (in g/L): total sugars, 48.11; total nitrogen, 1.05; total phosphorus, 0.43, and soluble proteins, 5.02 [1].

The realkalized fed-batch cultures with each LAB strain were carried out at a controlled temperature of 30°C in a 10 L bench top bioreactor tailored at an agitation speed of 200 rpm and continuous record of pH as described before [1, 21, 22].

 At the end of each realkalized fed-batch culture, the media were adjusted to pH 7.0 to facilitate the adsorption of the bacteriocin onto the producer strains [23]. Subsequently, the cultures (cells plus the fermentation products) were preserved at −20°C with skim milk powder (300 g/L of fermented medium) until further use, as indicated by Guerra et al. [1].

### 2.3. Preparation of the Experimental Feeds

The potentially probiotic preparations of the two LAB (composition in Table 1) were defrosted and mixed with the commercial mash broiler feed (named basal diet which composition is showed in Table 2) using an end-over-end mixer in a ratio of 20 mL probiotic preparation/kg feed. No pellet was made with the experimental feeds. This can contribute to increase feed wastage and, so, to increase feed conversion values; however for comparative purposes between treatments it is worth keeping high probiotic counts. The probiotic preparations were added weekly to the basal diets as recommended by Guerra et al. [1]. In the group receiving the antibiotic, the basal diet was supplemented with 10 mg of avilamycin per kg of feed.

### 2.4. Analytical Determinations

The concentrations of total sugars, phosphorous, nitrogen, protein, lactic acid, formic acid and antibacterial activity in the probiotic preparations were determined by methods previously described [23, 24].

### 2.5. Experimental Animals and Management

The two experiments were carried out in the experimental farm of COREN, S.C.L. (Ourense, Spain). One-day-old males were used in both experiments and fed *ad libitum*. Four experimental groups were assayed: a control group fed with unsupplemented basal diet, a second group fed with the probiotic *Lactobacillus casei *preparation, a third group fed with the probiotic *Lactococcus lactis* preparation, and an antibiotic (avilamycin) supplemented fed group.

#### 2.5.1. Experiment  1

A total of 120 medium-growth Sasso X44 chickens were distributed into 12 replicates of 10 birds each (three replicates per treatment) during an experimental period of 42 days. Each replicate was housed separately in individual metal cages (75 × 40 × 105 cm), situated at 80 cm from the floor to limit the consumption of faeces. The cycle of light was natural environment (12 h of daylight) and the temperature during the treatment, maintained with propane heating, was the following: first week, 32°C, second week, 30°C, and third week, 26°C. From day 21, the temperature was maintained between 20 and 24°C. The BWG, FI, and FCE were calculated at 7, 14, 21, and 42 days after the animals received the experimental diets.

#### 2.5.2. Experiment  2

A total of 1200 commercial Ross 308 broiler chicks were distributed into 24 pens (12.7 animals/m^2^) of 50 birds (six replicates per treatment) during an experimental period of 31 days on wood shaving floor to simulate farm conditions. According to the good manufacturing practices of Coren S.C.L., the light program consisted in 24 h for the first 12 days, one hour alternating light and dark from day 15 to day 21, and 2 h alternating light and dark from day 21 until the end of the experiment. The temperature program was similar to that of experiment 1. BWG, FI, and FCE were determined at 16 and 31 days.

### 2.6. Sample Collection

At 7, 14, 21, and 42 days (in case of experiment 1) and at 7, 16, and 31 days (in case of experiment 2), 24 chickens (2 per replicate, so 6 per treatment) in experiment 1 and 48 chickens (2 per replicate, so 12 per treatment) in experiment 2 were chosen randomly and sacrificed. Animal management followed the animal care and welfare guidelines of COREN S.C.L. The abdominal cavity was opened and the gastrointestinal tract was excised to determine the number of bacterial counts. In experiment 1 the whole gastrointestinal tract, from crop to caeca, was used. In experiment 2 only the caeca were excised.

### 2.7. Bacterial Counts in Gastrointestinal Samples

All gastrointestinal samples emptied of faeces were weighed and placed in a sterile bag with sterile phosphate buffered saline (PBS, pH 7, 100 mM NaCl) in proportion 1 : 10 (w/v) and pummelled for 2 min in a Stomacher. Tenfold serial dilutions in sterile PBS were performed up to 10^7^ and aliquots (0.1 mL) of each dilution were pour-plated in MRS agar (Cultimed), eosin methylene blue agar (EMB, Levine Formulation, Cultimed), and Tryptone Soy Agar (TSA, Cultimed) to determine the main representative cultivable LAB microbiota, coliforms, and total mesophilic counts, respectively. The plates were incubated at 37 ± 1°C for 24 h for coliform counts and at 30 ± 1°C for 24 h and 48 h for mesophilic bacteria and LAB counts, respectively. The results were expressed as the number of colony-forming units per gram (wet weight) of intestinal content (CFU/g).

### 2.8. Statistical Analysis

Data on growth performance, bacterial counts, and relative intestine weight were statistically analyzed using the software package SPSS 13.0 for Windows (Release 13.0.1; SPSS Inc., Chicago, IL, 2004). Viable counts in the intestinal content were transformed by logarithm (log_10_) before statistical analysis. Normal distribution of data as well as the independence and homogeneity of variances among treatment groups was verified by looking at the distribution of the data and the Fisher *F*-test (which is included in the *t*-test output), respectively. A one-way analysis of variance (ANOVA) with the step-down multiple-stage *F *post hoc test (Ryan-Einot-Gabriel-Welsch multiple *F*-test (*P* = 0.05) was used to distinguish treatment mean differences [1].

## 3. Results

### 3.1. Probiotic Preparations

The mean compositions of the two probiotic preparations obtained from the realkalized fed-batch fermentations with *Lact. casei *and* L. lactis* on whey are shown in Table 1. As it can be observed, both probiotic preparations contained relatively high concentrations of inhibitory products (lactic acid and antibacterial activity) and relatively low concentrations of formic acid, which were accumulated at different concentrations in the culture media during the realkalized fed-batch fermentations.

In addition, both preparations were characterized with high concentrations of viable cells (3.69 × 10^9^ CFU/mL in case of *Lact. casei* and 3.34 × 10^9^ CFU/mL in case of *L. lactis*). Therefore, addition of the two potential probiotic preparations at the dose of 20 mL/kg of feed offered the possibility of preparing feeds with viable cells concentrations of 7.38 × 10^7^ CFU of *Lact. casei* or 6.68 × 10^7^ CFU of *L. lactis* per g of feed. Both concentrations are higher than that of the recommended dose of viable probiotic cells (10^6^ CFU per g or mL) necessary to observe beneficial effects [25, 26].

### 3.2. Performance of Medium-Growth Sasso X44 Chickens in Cages (Experiment  1)

The BWG, FI, and FCE values of medium-growth Sasso X44 chickens fed with the different diets during the experimental period are shown in Table 3. Broilers fed the *L. lactis* CECT 539 preparation showed the lowest values of BWG and FI during the first 21 d of treatment (*P* < 0.05), but no differences were found for FCE among groups. In addition, for the entire experimental period, diets did not affect any of the growth performance parameters studied.

Interestingly, at the end of the experiment (42 d), avilamycin feeding resulted in a reduction (*P* < 0.05) in the mean relative intestinal weight (0.040 g of organ/g of BW) in comparison to the control group (0.046 g of organ/g of BW) and the groups receiving *Lact. casei* (0.045 g of organ/g of BW) and *L. lactis* (0.048 g of organ/g of BW). However, no significant differences in the relative intestine weight were observed between the two groups receiving the two probiotic preparations and the control group (Table 3).

### 3.3. Effect of the Feeding with Probiotic Preparations on the Intestinal Microbiota of Medium-Growth Sasso X44 Chickens in Cages (Experiment  1)

**Figure 1:**
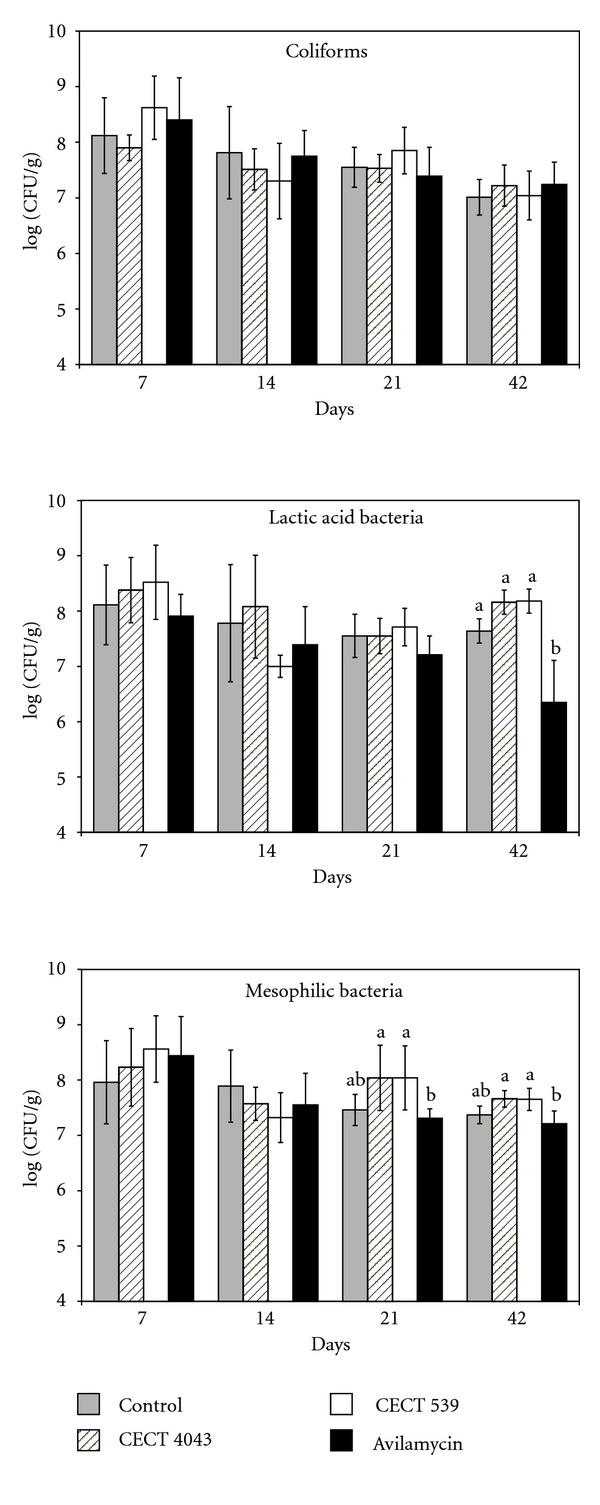
Viable plate counts (means ± standard deviations) of coliforms, lactic acid bacteria and mesophilic bacteria in the intestinal content of medium-growth Sasso X44 chickens fed with a non supplemented diet (control), or diets supplemented with the probiotic *Lact. casei *CECT 4043 or *L. lactis *CECT 539 preparations or avilamycin. ^a–c^Means within columns followed by different letters are significantly (*P* < 0.05).

The viable counts of the different groups of bacteria analyzed in this trial are shown in Figure 1. With regard to the coliforms (upper part of Figure 1), no significant differences were found in total counts among diets, probably because birds were placed in cages elevated 80 cm above the barn floor, where no reconsume of faeces occurred.

However, in broilers fed the probiotic preparations, the final average LAB counts (8.16 log_10_ CFU/g in case of strain CECT 4043 and 8.18 log_10_ CFU/g in case of strain CECT 539) were higher (*P* < 0.10) than that (7.64 log_10_ CFU/g) of the control group (middle part of Figure 1). In chickens fed avilamycin, the average LAB counts decreased progressively until reaching a final value at the end of the experiment (6.35 log_10_ CFU/g) significantly lower (*P* < 0.05) than those final levels obtained in the other three groups (middle part of Figure 1). 

As expected, the final average mesophilic counts in the groups fed the probiotic preparations (7.66 log_10_ CFU/g in case of strain CECT 4043 and 7.65 log_10_ CFU/g in case of strain CECT 539) by day 42 were significantly higher (*P* < 0.05) than that (7.21 log_10_ CFU/g) of the avilamycin group (lower part of Figure 1).

### 3.4. Performance of Broiler Chickens in Pens with Wood Shaving Litter and Subjected to Nutritional Stress (Experiment  2)

Table 4 summarizes the effect of dietary probiotic preparations or avilamycin on growth performance parameters in stressed chickens. With regard to the final BWG, no significant differences were observed among treatments during the whole experimental period. However, broilers fed avilamycin had higher FCE values (*P* < 0.05) than the other three groups. At 16 days of treatment, the FCE values in the groups fed the probiotic preparations (1.86 ± 0.08 in case of strain CECT 4043 and 1.82 ± 0.08 in case of strain CECT 539) were lower than those of the avilamycin (2.15 ± 0.18, *P* < 0.05) and control (2.04 ± 0.20) groups. Although the FCE values increased in the four groups at the end of the experimental period, broilers fed *Lact. casei *CECT 4043 preparation were more efficient than those fed avilamycin (*P* < 0.05) or unsupplemented feed (*P* < 0.10). In the present study, mortality was not significantly (*P* < 0.05) affected by probiotic treatment over the experimental period (data not shown).

Although, at the end of the experiment, the relative weight of caeca values in all the groups were approximately 1% of the animal weight, as described by Redig [27], no significant differences were found between broilers fed with the different diets.

### 3.5. Total Coliform Counts in the Caeca of Ross 308 Broiler Chickens (Experiment  2)

**Figure 2:**
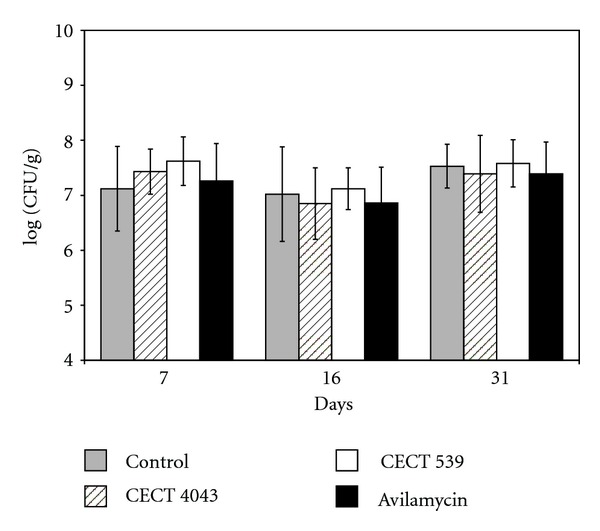
Viable plate counts (means ± standard deviations) of coliforms in the caeca of Ross 308 broilers fed with a non supplemented diet (control) or diets supplemented with the probiotic *Lact. casei *CECT 4043 or *L. lactis *CECT 539 preparations or avilamycin.

Coliform counts in the caeca were highly variable throughout the assay, with large variance of values within individual treatment groups and variations observed in counts at different time points (Figure 2). Consequently, no significant difference in fecal coliform counts (*P* < 0.05) was observed in the four groups during the experimental period.

## 4. Discussion

The results obtained in experiment 1 showed that, at the end of the experimental period (42 days), consumption of the different diets did not affect any of the growth performance parameters of medium-growth Sasso X44 chickens. However, a significant decrease in the relative intestinal weight (*P* < 0.05) was observed in the group receiving avilamycin as compared with the nontreated control group and the groups fed diets with the probiotic preparations. A similar result was observed when bacitracin and virginiamycin, two antibiotic growth promoters, were assayed as additives in corn-soybean meal diets for Ross × Ross broiler chicks [28]. This decrease in the intestinal weight because of consumption of antibiotics has been documented by other authors [29, 30] before knowing their positive effect as growth promoters in chicks. Reduction in intestinal weight has been associated to a thinning of the gastrointestinal walls tract probably due to an inhibition of the microbial production of polyamines and volatile fatty acids [31].

Interestingly, in the present study, the groups receiving the two probiotic preparations had similar relative intestine weights than the group fed control diet (Table 3), suggesting that the mechanism of action of avilamycin and the probiotic preparations was different.

In the first experiment, no significant differences were found in total coliform counts among groups (Figure 1). In this way, when a pathogen colonizes the intestine, infection in the other chickens happens by horizontal transmission through faeces [32], but, in this assay, chickens had a minimum contact with their faeces and, consequently, the growth of enteric bacterial contamination was avoided.

At the end of the experimental period, LAB counts in the probiotic fed groups were higher than that of the control group. The strains CECT 4043 and CECT 539 used in this experiment have proven to be resistant to acid and bile salts *in vitro *under conditions that mimic the animal GIT environment [1]. Therefore, the higher LAB counts found in chickens fed probiotics could be due to the presence of the *Lact*. *casei* CECT 4043 and *L. lactis* CECT 539 in the GIT, permanently as a result of the colonization of the intestinal mucus, or temporarily and dependent on the diet consumed by the birds. Another possible reason to explain the increase in LAB counts could be the growth of other epiphytic LAB due to the probiotic cells supplemented in the diet. Contrarily, chickens fed with avilamycin showed a continuous decrease in LAB counts, which were at the end of the experiment (42 days), significantly lower than those of the groups receiving the two probiotic preparations. These results suggest that the feeding with avilamycin inhibits the development of LAB. Also the highest counts of mesophilic bacteria were found in chicks fed the two probiotic preparations. As mesophilic bacteria also include LAB, the increase in mesophilic counts observed in the probiotic groups could be due to the same reasons previously discussed for LAB counts. Yu et al. [33] also reported that Arbor Acres broiler fed a *Lactobacillus reuteri* Pg4 transformant in pens showed higher total aerobic and *Lactobacillus* spp. counts in the ileum and caecum than unsupplemented control birds. In conclusion, the experimental system chosen in this first trial for the handling of animals, which reduces their contact with faeces, could mask a potential protective effect of probiotic or antibiotic. These results suggested the need of choosing other handling conditions.

Then, in experiment 2, the effect of the probiotic preparations containing *Lact. casei* CECT 4043 and *L. lactis *CECT 539 cultures was evaluated in wood shaving floor, using the similar conditions of temperature, cycle of light, and handling as the farm conditions. With this approach, the transmission of microorganisms between animals could be facilitated. The medium-growth Sasso X44 chickens previously used in the first trial were replaced by Ross 308 broilers in the second trial. In order to obtain a more accurate analysis, both the number of animals per treatment and the number of replicates were increased. Additionally, a feed based on barley and wheat was used to promote intestinal adverse conditions that could be reversed or improved by using a probiotic treatment. These cereals contain high amounts of nonstarch polysaccharides (NSPs), which have “anti-nutritive effects” and increase the viscosity of the intestinal content [34]. The use of barley can deteriorate the intestinal structure, causing decrease of length and width of villi and their atrophy [35, 36]. Otherwise, Hofshagen and Kaldhusdal [37] observed that the counts of *Lactobacillus* and *Streptococcus* strains were higher in chickens fed with diets based on corn than chickens fed diets with barley. Moreover, Dalloul et al. [38] observed an increase in resistance to coccidiosis in chickens fed with a *Lactobacillus*-based probiotic diet. These results support the hypothesis that these bacteria could control the necrotic enteritis and it could be expected that the probiotic feed used in this trial would protect against this infection.

In this second experiment the FCE values of broilers fed with *Lact. casei *CECT 4043 preparation were lower (*P* < 0.10) than that of the broilers fed with the control diet at 16 and 31 days. With respect to avilamycin, the decrease of FCE was statistically significant (*P* < 0.05) in both periods. This positive effect of *Lactobacillus-*supplemented diets on FCE has been previously reported. Thus, laying hens, that received *L. acidophilus* supplemented diets for a 48-week period [39], had significantly better FCE than control birds. In the same way, the addition of either the single *L. acidophilus* or a mixture of 12 *Lactobacillus* cultures significantly improved FCE in broilers [14].

The use of the wheat and barley diet did not increase mortality in any of the treatments. Thus, in the second experiment, slighter and subclinic necrotic enteritis could occur, as this type of enteritidis is asymptomatic [40]. Consequently, it is probable that the nutritional stress induced in this trial was not enough to see the growth-stimulating effects of the probiotic bacteria in animal exposed to stress reported by other authors [41, 42].

In experiment 1, no significant differences were found between treatments in total coliform counts in the whole intestinal tract (Figure 1). However, it has been reported that bacterial distribution along the gastrointestinal tract is not uniform [33]. The high acidity in the stomach as well as the concentration of bile components in the proximal intestine determines a strain selection [43] and reduces the bacteria counts in the proximal segments of intestine, crop, and ileum, in comparison with the caecum. But the homogenization of the contents of the whole intestine could minimize differences among the different segments. In this manner, Jin et al. [14] did not observe significant differences in the coliform population in the small intestine of broilers fed diets supplemented with *Lactobacillus* during the experimental period, although the number of coliforms was significantly reduced in the caeca. For these reasons, and considering that the caecum is a distal part where more favourable conditions for bacterial development exist, in experiment 2, this part was used to analyze the number of total coliform counts. However, no significant differences (*P* < 0.05) were found in total coliform counts between the treatments (Figure 2).

Probiotics inhibit the adhesion of certain pathogenic bacteria such as *E. coli* and *Salmonella enterica* to the epithelial cells *in vitro* [44]. Competitive binding to receptors or the stimulation of host factors such as the production of mucin has been proposed as possible reasons to explain this inhibition [45]. However, not always inhibitory effects of probiotic strains on the growth of coliforms are observed in *in vivo* trials because host-dependent mechanisms are important in reducing the coliform level [46]. Guerra et al. [1], reported that viable coliform counts in pigs fed *L. lactis *CECT 539 and *Lact*. *casei *CECT 4043 preparations dropped on average for 1.8 and 1.4 log units, respectively mean; while viable coliform counts did not change in the control group. However, in the current experiment, the above discussed coliform counts reduction was not observed. The host-dependent theory suggested by Meimandipour et al. [46] could be used to explain this fact.

On the other hand, when *Lact*. *casei *CECT 4043 preparation was tested as probiotic in pigs, the mean final BWG and FI values were higher than those observed in the control group [1]. However, these researchers did not observe significant differences in FCE between the two groups receiving probiotic preparations and the control group. In contrast, the results of present study showed that, by day 31, the final BWG values in chickens receiving the different diets were not significantly different (Table 4), but the animals fed *Lact. casei *CECT 4043 preparation improved their final FCE in comparison to chickens fed avilamycin.

However, it is worth highlighting that differences usually found in BWG between chickens fed with antibiotics and chickens fed with diets without growth promoters were not present in this case (Tables 3 and 4). Patterson and Burkholder [10] recommended that studies in which there is no response to the growth promotant antibiotics should not be considered negative for the probiotic treatment. Then, the lack of differences between antibiotic and control groups in both experiments (medium-growth Sasso X44 chickens and Ross 308 broilers) suggests that the probiotic effect of *Lact. casei *CECT 4043 preparation observed in chickens has been of minor magnitude because of the good condition of the animals.

## 5. Conclusions

The ability of *Lact. casei *CECT 4043 to improve the FCE in chickens, together with its capability to stimulate the growth and to reduce coliform counts in the faeces of postweaning piglets, indicates that the probiotic *Lact. casei *CECT 4043 preparation could be successfully used as a feed additive for the animal feed industry. In addition the different probiotic effect observed in pigs and broilers supports the hypothesis that probiotic mechanisms are host dependent. The results obtained in this study reinforce previous reports on the probiotic effect of *Lactobacillus* sp. on chicken and pig growth.

## Figures and Tables

**Table 1:** Mean composition of the probiotic preparations obtained from realkalized fed-batch cultures of *Lact. casei *CECT 4043 and *L. lactis *CECT 539.

Composition	CECT 4043	CECT 539
Total sugars (g/L)	16.16	19.71
Nitrogen (g/L)	1.37	1.59
Phosphorous (g/L)	0.07	0.36
Protein (g/L)	6.09	9.15
pH	7.00	7.00
Viable cells (CFU/mL)^1^	3.69 × 10^9^	3.34 × 10^9^
Antibacterial activity (AU/mL)^2^	28.85	164.49
Lactic acid (g/L)	33.47	17.54
Formic acid (g/L)	0.10	0.85

^1^CFU: colony-forming units.

^2^AU: activity units.

**Table 2:** Composition of the basal diets of both experiments.

Composition (g/Kg of diet)	Experiment 1	Experiment 2
Barley	20.0	70.0
Wheat	300.0	500.0
Maize	300.0	0.0
Animal fat	15.0	48.2
Full fat soybean	35.0	28.6
Soybean meal 440	272.0	20.0
Gluten feed	20.0	0.0
Soybean meal 470	0.0	287.4
Sodium bicarbonate	0.3	0.5
Calcium carbonate	14.0	11.2
Monocalcium phosphate	13.5	19.6
Sodium chloride	3.9	3.5
L-Choline 75	0.9	0.9
DL(+)-Methionine	1.9	3.4
L-Threonine	0.0	0.6
L-Lysine	0.7	3.3
Coccidiostate^a^	0.6	0.6
Mineral premix^b^	1.1	1.1
Vitamin premix^c^	1.1	1.1

^a^C-Maxiban G150.

^b^Premix contained per kg of diet: Co 0.15 g, Cu 8 g, Fe 40 g, I 1.9 g, Mn 80 g, Se 0.25 g, Zn 65 g.

^c^Premix contained per kg of diet: Vitamin A 12000 IU, Vitamin D_3_ 4000 IU, Vitamin E 50 IU, Vitamin K 3.5 mg, Vitamin B_1_ 2.5 mg, Vitamin B_2_ 7 mg, pantothenic acid 14 mg, Vitamin B_6_ 3 mg, Vitamin B_12_ 15 mg, nicotinic acid 55 mg, folic acid 1 mg, biotin 0.17 mg.

**Table 3:** Effect of dietary probiotic preparations (CECT 4043, CECT 539) or antibiotic (avilamycin) on growth performance parameters of medium-growth Sasso X44 chickens during 42 days (experiment  1).

Treatment^1^	BWG (g per chicken)	FI (g per chicken)	FCE (g of FI/g of BWG)	Relative intestine weight (g of organ/g of BW)
Chicken performance (days 1–21)				

Control	436 ± 21^a^	749 ± 16^a^	1.72 ± 0.05	0.066 ± 0.006
CECT 4043	425 ± 5^ab^	754 ± 9^a^	1.78 ± 0.01	0.064 ± 0.003
CECT 539	395 ± 13^b^	654 ± 80^b^	1.65 ± 0.15	0.067 ± 0.007
Avilamycin	440 ± 17^a^	766 ± 15^a^	1.74 ± 0.05	0.060 ± 0.009
*F*	5.477	4.630	1.179	0.965
d.f. (*N*)^2^	3 (12)	3 (12)	3 (12)	3 (24)

Chicken performance (days 1–42)				

Control	1336 ± 33	2864 ± 140	2.14 ± 0.07	0.046 ± 0.004^a^
CECT 4043	1348 ± 33	2858 ± 97	2.12 ± 0.05	0.045 ± 0.002^a^
CECT 539	1314 ± 16	2800 ± 120	2.13 ± 0.07	0.048 ± 0.006^a^
Avilamycin	1503 ± 16	3084 ± 84	2.06 ± 0.14	0.040 ± 0.004^b^
*F*	3.935	3.741	0.584	4.242
d.f. (*N*)	3 (12)	3 (12)	3 (12)	3 (24)

^a–c^Means within columns followed by different letters are significantly different (*P* < 0.05).

^1^The chickens from the control group were not given probiotic preparations or antibiotic. The chickens in the CECT 4043, CECT 539, and avilamycin groups were fed with *Lactobacillus casei *CECT 4043 (7.38 × 10^10^ CFU/Kg diet), *Lactococcus lactis *CECT 539 (6.68 × 10^10 ^CFU/Kg diet) preparations, and avilamycin (10 mg/Kg diet), respectively. BWG: body weight gain, FI: feed intake, FCE: feed conversion efficiency.

^2^d.f.: degree of freedom. *N*: number of samples.

**Table 4:** Effect of dietary probiotic preparations (CECT 4043, CECT 539) or antibiotic (avilamycin) on growth performance parameters of Ross 308 broiler chickens subjected to nutritional stress (experiment  2).

Treatment^1^	BWG (g per chicken)	FI (g per chicken)	FCE (g of FI/g of BWG)	Relative caeca weight (g of organ/g of BW)
Chicken performance (days 1–16)				

Control	397 ± 34	805 ± 42^a^	2.04 ± 0.20^ba^	0.012 ± 0.003
CECT 4043	387 ± 13	721 ± 9^b^	1.86 ± 0.08^cb^	0.011 ± 0.003
CECT 539	388 ± 15	706 ± 18^b^	1.82 ± 0.08^c^	0.011 ± 0.002
Avilamycin	369 ± 39	789 ± 47^a^	2.15 ± 0.18^a^	0.012 ± 0.003
*F*	1.025	13.042	6.810	1.669
d.f (*N*)^2^	3 (24)	3 (24)	3 (24)	3 (48)

Chicken performance (days 1–31)				

Control	1377 ± 82	2909 ± 154	2.11 ± 0.10^ab^	0.009 ± 0.003
CECT 4043	1388 ± 42	2802 ± 34	2.02 ± 0.05^b^	0.009 ± 0.003
CECT 539	1364 ± 46	2812 ± 56	2.06 ± 0.07^ab^	0.008 ± 0.003
Avilamycin	1319 ± 87	2872 ± 33	2.18 ± 0.12^a^	0.007 ± 0.003
*F*	1.221	2.135	3.689	3.446
d.f (*N*)	3 (24)	3 (24)	3 (24)	3 (44)

^a–c^Means within columns followed by different letters are significantly different (*P* < 0.05).

^1^The chickens from the control group were not given probiotic preparations or antibiotic. The chickens in the CECT 4043, CECT 539, and avilamycin groups were fed with *Lactobacillus casei *CECT 4043 (7.38 × 10^10^ CFU/Kg diet), *Lactococcus lactis *CECT 539 (6.68 × 10^10 ^CFU/Kg diet) preparations, and avilamycin (10 mg/Kg diet), respectively. BWG: body weight gain, FI: feed intake, FCE: feed conversion efficiency.

^2^d.f.: degree of freedom. *N*: number of samples.
